# No association between exacerbation frequency and stroke in patients with COPD

**DOI:** 10.2147/COPD.S95775

**Published:** 2016-02-02

**Authors:** Claire Windsor, Emily Herrett, Liam Smeeth, Jennifer Kathleen Quint

**Affiliations:** 1 Faculty of Epidemiology and Population Health, London School of Hygiene and Tropical Medicine, London, UK; 2 Department of Respiratory Epidemiology, Occupational Medicine and Public Health, National Heart and Lung Institute, Imperial College London, London, UK

**Keywords:** COPD exacerbations, stroke, frequent exacerbators, infrequent exacerbators

## Abstract

**Background:**

Patients with chronic obstructive pulmonary disease (COPD) have a higher risk of stroke than the general population. Chronic inflammation associated with COPD is thought to contribute to this risk. Exacerbations of COPD are associated with a rise in inflammation, suggesting that there may be an association between exacerbation frequency and the risk of stroke. This study examined that association.

**Methods:**

Using the UK Clinical Practice Research Datalink, COPD patients with a first stroke between January 2004 and December 2013 were identified as cases and matched on age, sex, and general practice to controls with COPD but without a stroke (6,441 cases and 19,323 controls). Frequent exacerbators (FEs) were defined as COPD patients with ≥2 exacerbations, and infrequent exacerbators (IEs) have ≤1 exacerbation in the year prior to their stroke. Conditional logistic regression was used to estimate the association between exacerbation frequency and stroke overall, and by stroke subtype (hemorrhagic, ischemic, or transient ischemic attack). Exacerbations were also categorized into 0, 1, 2, or ≥3 exacerbations in the year prior to stroke.

**Results:**

There was no evidence that FE had an increased odds of stroke compared to IE (OR [odds ratio] =0.95, 95% CI [confidence interval] =0.89–1.01). There was strong evidence that the risk of stroke decreased with each exacerbation of COPD experienced per year (*P*_trend_ =0.003). In the subgroup analysis investigating stroke subtype, FE had 33% lower odds of hemorrhagic stroke than IE (OR =0.67, 95% CI =0.51–0.88, *P*=0.003). No association was found within other stroke types.

**Conclusion:**

This study found no evidence of a difference in the odds of stroke between IE and FE, suggesting that exacerbation frequency is unlikely to be the reason for increased stroke risk among COPD patients. Further research is needed to explore the association through investigation of stroke risk and the severity, duration, treatment of exacerbations, and concurrent treatment of cardiovascular risk factors.

## Introduction

Chronic obstructive pulmonary disease (COPD) is characterized by inflammation of the small airways and lung parenchyma, which leads to progressive airflow limitation.^1^ By 2020, COPD is expected to be the third most common cause of death worldwide.^2^ Cardiovascular disease (CVD) is a major cause of morbidity and mortality in COPD patients, with lung cancer and CVD being the commonest causes of death in patients with moderate COPD.^1^,^3^

Stroke is the second leading cause of death worldwide and the most important cause of acquired disability in most parts of the world.^4^ The prevalence and incidence of stroke are higher in COPD patients than in the general population.^5^,^6^

Atherosclerosis is the common underlying pathological process in the development of the majority of ischemic strokes, with the strongest risk factors for stroke of any type being hypertension and smoking.^4^ Local lung and systemic inflammation have been shown to accelerate the process of atherosclerosis, and inflammation is an independent risk factor for CVD.^1^–^4^ Not only is COPD itself a raised inflammatory state, exacerbations of COPD are associated with acutely increased periods of both lung and systemic inflammation.^7^ The resulting acute inflammatory changes may correspond to a temporary rise in the risk of stroke. In a self-controlled case series of 25,875 COPD patients carried out within a UK primary care database, there was a 1.26-fold (95% CI [confidence interval] =1.0–1.6) increased risk of stroke 1–49 days after an exacerbation.^8^ Frequent exacerbators (FEs) of COPD,^9^ people who have two or more health care utilization exacerbations per year, have increased airway and systemic inflammation, even in the stable state. They also have been shown to have increased arterial stiffness,^10^ which is a validated, independent risk factor for cardiovascular events.^11^ While there is no published literature to date on exacerbation frequency and risk of stroke, there are at least two potential mechanisms by which FE could have an increased risk of stroke, and there is evidence that history of stroke is more common in FE.^12^

Using data from the Clinical Practice Research Datalink (CPRD), we undertook a matched case control study to determine if the risk of stroke varied within COPD patients according to the number of exacerbations they experienced. Our specific objectives were: 1) to estimate the association between exacerbation frequency and stroke risk, 2) to determine if there was a dose–response relationship between the number of exacerbations per year and the risk of stroke, and 3) to determine if this varied by stroke subtype; hemorrhagic, ischemic, or transient ischemic attack (TIA).

## Methods

### Data source

CPRD is the world’s largest validated computerized database of anonymized longitudinal medical records for primary care.^13^,^14^ Data comprise approximately 14 million patients from 660 primary care practices spread throughout the UK. Records are derived from a widely used general practice (GP) software system and contain complete prescribing data, and Read-coded diagnostic and clinical information as well as information on tests requested, laboratory results, and referrals made at or following on from each consultation.^15^ The population of patients within CPRD have been shown to be representative of the UK population with respect to age, sex, and geographical distribution.^16^

### Study population

The study population consisted of patients over 35 years old, with a validated diagnosis of COPD.^17^

### Primary outcome

Cases were all individuals in the study population who experienced a stroke between January 1, 2004 and December 31, 2013. Validated diagnostic Read codes were used to define stroke.^18^ The primary outcome was created by grouping any event with a diagnostic Read code for ischemic/hemorrhagic/nonspecific stroke or TIA. The secondary outcome was type of stroke, which was coded as either ischemic, hemorrhagic, or TIA. Patients were excluded if they had a previous stroke or TIA in their CPRD record, were not registered with CPRD at the time of their stroke, or had less than 1 year of standard follow-up prior to the date of stroke.

Three controls per case were selected from the study population, on the date of the stroke in the case (index date). Controls were matched to cases for age (within 5 years), sex, and GP. Inclusion and exclusion criteria for the controls were the same as those for cases (using index date instead of stroke date to define eligibility). Controls were eligible to become cases later during their follow-up and were allowed to be used as a control for more than one case.

### Defining COPD exacerbations

An exacerbation of COPD was defined using a validated definition as an episode that was recorded in CPRD using Read codes as an acute exacerbation of COPD code or a lower respiratory tract infection (LRTI) code where antibiotics and/or oral steroids were also issued on the same day as the LRTI code.^19^

The number of exacerbations in the year prior to the index date was categorized in two ways. For the first analysis, “frequent exacerbators” were defined as individuals with ≥2 recorded exacerbations in the year prior to the index date, while “infrequent exacerbators” were individuals who had ≤1 exacerbation in the year prior to the index date. Second, exacerbation frequency was grouped into 0, 1, 2, or ≥3 exacerbations in the previous year.

### Covariates

Possible confounders were extracted from CPRD and included demographic factors (age [categorized into four age-bands; 35–59, 60–69, 70–79, and ≥80 years], sex, ethnicity, BMI [body mass index; using World Health Organization classification]), comorbid cardiovascular disease (angina, heart failure, previous coronary intervention [percutaneous coronary intervention or coronary artery bypass graft {CABG}], peripheral arterial disease, acute coronary syndrome, myocardial infarction, angina), stroke risk factors (hypertension, diabetes, dyslipidemia, family history of cardiovascular or cerebrovascular disease, smoking history [current, ex, or never], atrial fibrillation), and drug prescriptions (antiplatelets, anticoagulants, statins, angiotensin-converting enzyme inhibitors, angiotensin receptor blockers, nitrates, calcium channel blockers, β-blockers). For all medications, a patient was considered exposed if their record contained at least two prescriptions of the same medication prior to the index date. The Global Initiative for Chronic Obstructive Lung Disease (GOLD) 2011 staging system was used to define COPD severity with respect to airflow limitation.^20^ Code lists for all covariates are available on request.

### Statistical analysis

Conditional logistic regression was used to compare the odds of stroke in “frequent” and “infrequent” exacerbators. Causal modeling was performed using DaGitty software.^21^ Smoking status was an a priori confounder, and age and sex were considered as a priori effect modifiers. Any variable which was related to both COPD exacerbations and stroke was included. Other variables were added sequentially. Variables taken forward to the full model were identified using a causal modeling approach, clinical knowledge, or they were identified from the univariable analysis, having been strongly associated with stroke, or deemed of sufficient priori interest to include. Conditional logistic regression, including interaction terms, was used to assess interaction between exacerbation frequency and age, as well as exacerbation frequency and sex. To examine the association between exacerbation frequency and stroke subtype, we repeated the analysis in a dataset restricted to 1) ischemic stroke, 2) hemorrhagic stroke, and 3) TIA. For each subtype we performed conditional logistic regression, adjusting for the confounding factors identified in the primary analysis. Finally, to explore a dose–response relationship between exacerbation frequency and stroke, we repeated the primary analysis using exacerbation frequency defined as 0, 1, 2, or ≥3 exacerbations in the year prior to index date.

### Sensitivity analysis

To explore the impact of missing data on the analysis, a sensitivity analysis was carried out for variables with large amounts of missing values. Fully adjusted conditional regression models with and without the variable were compared in complete case analyses.

### Power calculation

The prevalence of FE among all COPD patients was 29% in a previous UK study.^9^ Taking this value as the estimated prevalence of FE in the controls, with 6,441 cases and 19,323 matched controls, this study would have a power of greater than 90% to detect a minimum odds ratio (OR) greater than 1 (1.11) and a maximum OR less than 1 (0.9) with an α of 0.05.

### Ethics

Ethical approval was obtained from the local LSHTM ethics committee (ref 7448) as well as the Independent Scientific Advisory Committee (ISAC), (protocol 14_100).

## Results

The final dataset consisted of 6,441 cases and 19,323 controls. The study population consisted of more men than women (53.9% vs 46.1%). The median age of the population was 76 years (interquartile range: 69–82). Approximately 26.8% (1,723/6,441) of cases were FE compared to 28% (5,430/19,323) of controls (OR =0.93, 95% CI =0.87–0.99). Hypertension, smoking, dyslipidemia, and diabetes were all strongly associated with increased odds of stroke (Table 1). There were also strong associations between stroke and peripheral arterial disease, heart failure, atrial fibrillation, and previous use of cardiac medications (Table 1). There was a strong association between GOLD stage and stroke (*P*=0.002), with the odds of stroke decreasing with increasing COPD grade severity.

### Association between exacerbation frequency and all stroke type

Approximately 26.8% (1,722/6,431) of cases were FE compared to 28.1% (5,428/19,292) of controls. There was no difference in the odds of stroke after adjusting for matched variables and smoking in FE and IE; (OR =0.94, 95% CI =0.88–1.00). After adjusting for age, sex, GP, smoking status, diabetes, hypertension, dyslipidemia, heart failure, CABG prior to stroke, atrial fibrillation, peripheral arterial disease, β-blockers, and calcium channel blockers, there was no evidence of an association between exacerbation frequency and the odds of stroke (OR =0.95, 95% CI =0.89–1.01, *P*=0.09) (Table 2). There was no evidence that the association between exacerbation frequency and stroke varied with age (LRT [likelihood ratio test] for interaction *P*=0.11) or sex (LRT for interaction *P*=0.57).

#### Dose–response relationship between number of exacerbations and risk of stroke

**Figure 1:**
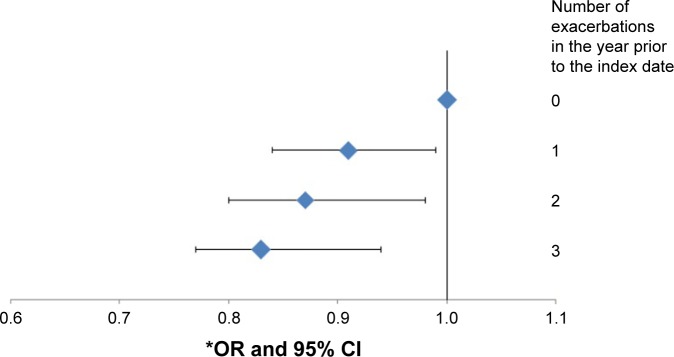
Association between number of COPD exacerbations per year and stroke. **Notes:** ^*^Adjusted for age, sex, GP, smoking, hypertension, diabetes, dyslipidemia, atrial fibrillation, CABG, PAD, heart failure, calcium channel blockers, β-blockers. *P*_trend_ =0.003. **Abbreviations:** OR, odds ratio; CI, confidence interval; COPD, chronic obstructive pulmonary disease; GP, general practice; CABG, coronary artery bypass graft; PAD, peripheral artery disease.

There was evidence of an association between number of exacerbations (grouped as 0, 1, 2, or ≥3 exacerbations in the year prior to index date) and stroke (all subtypes). Patients with three or more COPD exacerbations in the year prior to index date, had 15% lower odds of stroke than patients with no exacerbations (OR =0.85, CI =0.77–0.94). There was strong evidence for a linear trend after adjusting for confounders (*P*_trend_ =0.003). The odds of stroke decreased by approximately 5% with each additional exacerbation per year (OR =0.95, 95% CI =0.92–0.98) (Figure 1).

### Association between exacerbation frequency and stroke subtypes

In a dataset limited to patients where cases had hemorrhagic stroke (n=420 cases, 1,260 matched controls), 21.2% (89/420) of cases were FE compared to 28.6% (360/1,260) of controls.

In multivariable analysis, there was strong evidence of an association between exacerbation frequency and hemorrhagic stroke. Patients with frequent exacerbations had 33% lower odds of hemorrhagic stroke than patients with infrequent exacerbations (OR =0.67, 95% CI =0.51–0.88). There was no evidence for an association with either ischemic stroke or TIA and frequent exacerbations (Table 2).

### Sensitivity analysis

With respect to missing data, 13.6% (3,492/25,764) of observations had missing data on GOLD staging, 3% (765/25,764) had missing data on BMI status, and 0.2% (41/25,764) had missing data on smoking status (Table S1). For all three variables, individuals with missing data were more likely to be over 80 years old, than individuals without missing data.

In a complete case analysis including adjustment for GOLD stage, there was no evidence of an association between exacerbation frequency and stroke (adjusted OR =0.96, 95% CI =0.90–1.03), the OR including GOLD stage was almost identical to the main analysis (crude OR =0.94, 95% CI =0.88–1.00, adjusted OR =0.95, 95% CI =0.89–1.01, *P*=0.09).

## Discussion

Our analysis showed that FE of COPD had similar odds of stroke to IE. However, on further exploration of the association between exacerbations and stroke, we found the odds of stroke decreased by approximately 5% with each additional exacerbation per year. When investing the association between exacerbation frequency and risk of each stroke subtype, there was strong evidence that FE had 33% lower odds of hemorrhagic stroke than IE (OR =0.67, 95% CI =0.51–0.88, *P*=0.003). However, there was no evidence for an association with either ischemic stroke or TIA.

The reason we found no association between exacerbation frequency and risk of stroke, but a strong inverse association between exacerbation number and risk of stroke is likely due to the way in which exacerbation frequency was defined. In our primary analysis, we split the COPD patients into two groups (≥2 exacerbations vs ≤1 exacerbation) such that the baseline group included patients with an exacerbation. In our secondary analysis, we split COPD patients into groups by exacerbation number (0, 1, 2, 3+), which allowed comparison to patients with no exacerbations as baseline.

Our findings are somewhat contradictory to a previous study which found increased stroke risk acutely after an exacerbation.^8^ However, that study only quoted risk for ischemic strokes/TIA, and the association was only significant when exacerbations were defined as treatment with antibiotics (not with steroids alone or both). Additionally, the preferred definition of an exacerbation was not prestated, meaning that the study may have been more prone to Type-1 error. Another self-controlled case series set in primary care database found an approximately threefold increase in incidence rate of stroke in the first 3 days following an acute respiratory tract infection (IR =3.19, 95% CI =2.81–3.62) in the general population.^22^ This is in-keeping with the Donaldson et al study,^8^ although the at-risk period for stroke following a respiratory tract infection was much shorter than the at-risk period found following a COPD exacerbation, and the strength of the association was greater.

There are two plausible reasons why a COPD patient with increasing numbers of exacerbations may have a lower risk of stroke. The first is that a treatment for exacerbations causes a reduction in stroke risk. Oral glucocorticoids have been shown to reduce stroke risk in other studies, both in patients with COPD and in patients with rheumatoid arthritis.^23^ Our study was unable to explore the effect of steroids, as taking steroids was part of the exposure definition, making the effects of the two impossible to disassociate. Equally, the use of inhaled corticosteroids, which are more commonly prescribed for FE, may offer a protective effect. This warrants further investigation. A second explanation is that by definition, each exacerbation resulted in contact with health care services, meaning that those with more exacerbations are seen or reviewed more regularly by a health care professional. This may result in better management of other risk factors for stroke, including hypertension, smoking, and diabetes, for which there may have been residual confounding. While it is known that exacerbations are associated with increased mortality and therefore it could be argued that our findings are due to a healthy survivor effect, given that previous studies have shown associations between respiratory infections/exacerbations and stroke and the fact that FE exist across all stages of COPD severity, this is unlikely.^8^,^22^ The disproportionate reduction in the risk of hemorrhagic stroke, compared to other stroke types, may reflect the importance of hypertension as a risk factor for hemorrhagic stroke. The impact of more frequent contacts with the GP and better opportunistic checking of blood pressure, in patients with frequent exacerbations, may lead to better hemorrhagic stroke prevention than other subtypes. This merits further investigation.

Two large, key epidemiological studies have demonstrated an increased risk of stroke in COPD patients, compared to the general population.^5^,^6^ The first was an historical cohort study using data from patients registered in the Kaiser Permanente Medical Care Program.^5^ COPD patients had a higher risk of both hospitalization and death due to CVD (a composite measure, which included stroke). The risks were lower when looked at for stroke as an individual endpoint, and the association with stroke was weaker than all other measured endpoints (adjusted RR of death due to stroke was 1.25, CI =1.03–1.51). The study included 45,966 COPD cases, followed up for 4 years, which was a key strength; however, there was no adjustment for smoking, and therefore residual confounding is likely to be a source of bias. The second study was a cross-sectional and cohort analysis comparing the prevalence and risk of CVD in patients with COPD to those without in a British primary care database.^6^ COPD patients had higher odds of prevalent CVD (OR =4.98, 95% CI =4.85–5.81) and stroke (OR =3.34, 95% CI =3.21–3.48), as well as higher risk of incident CVD and stroke. In this study, CVD was a composite endpoint not including stroke. Despite this strong evidence for increased stroke risk in COPD patients, our study has shown that exacerbation frequency is unlikely to be driving the increased risk of stroke among COPD patients.

### Strengths

A key strength of our study was the size of the dataset; there was sufficient power to detect very small differences for the primary outcome. Matching on age, sex, and practice enhanced the precision of the estimated effects and allowed for some control of unmeasurable confounders related to which practice a patient belonged to, such as ease of access and prescribing practices.

The use of the specific diagnostic Read codes to identify patients with COPD has recently been validated.^24^ The definition of acute exacerbations of COPD has also been validated.^19^ A recent validation study of stroke codes used in CPRD showed that the positive predictive value of 75 identified stroke codes was 89% (95% CI =87%–91%).^18^ Data recorded in CPRD has been shown to be representative of patients throughout the UK. Another strength of this study is that it is community based. Most patients with COPD are treated in the community, and most exacerbations of COPD are mild to moderate in severity. This means that the findings are generalizable to the majority of COPD patients treated in the community, across the UK.

### Limitations

All cases that occurred over the study period, in participating practices, were included, provided that they had presented to health services and been diagnosed as having a stroke. Some strokes may have occurred in patients who did not present to health care services; however, given the symptoms involved in a stroke, this is not likely to be a large number of cases. Some strokes may have resulted in death and not have been recorded with a diagnostic code in the medical records, but again this is likely to be a very small number. There was no information on how such missed cases would have differed from identified cases with respect to their exposure status; however, the overall impact in terms of bias is likely to be small.

Any health care utilization definition will miss unreported exacerbations. Exacerbations may also be misdiagnosed as other conditions with similar clinical presentations such as heart failure and pulmonary embolism. It is therefore likely that the number of exacerbations recorded in this study is an underestimate of the total number exacerbations experienced, which would lead to an underestimate of the association between frequent exacerbations and stroke.

Residual confounding could have resulted from unmeasured, incomplete measuring, or misclassification of confounders. Unmeasured risk factors for stroke include regular physical activity, alcohol intake, depression, and psychological stress.^4^ Data for physical activity and alcohol intake were not extracted from CPRD because they were not recorded routinely. It would have been useful to look at the confounding effect of depression and psychological stress, since both are more common in patients with chronic diseases, such as COPD, and may be increased in patients with frequent exacerbations.

The validity of the use of Read codes to define smoking is lower than the use of codes to define specific diagnoses, such as stroke or COPD.^25^ Current smoking tends to be well recorded, but previous smoking is not well recorded. In clinical practice, ex-smokers tend to be misclassified as never smokers. This would lead to an underestimate of the number of ex-smokers, meaning that the OR for stroke were not fully adjusted for the effect of smoking.

## Conclusion

This study found no evidence of a difference in the odds of stroke between COPD patients with low exacerbation frequency (0 or 1 per year) and those with high exacerbation frequency (2 or more per year), and therefore exacerbation frequency is unlikely to be the reason for increased stroke risk among COPD patients. Furthermore, there was a decreased risk of stroke in COPD patients with an increasing number of exacerbations. However, this association is likely to be more complex than exacerbation numbers alone and we recommend further exploration of this association through investigation of stroke risk and the severity, duration, treatment of exacerbations, and concurrent treatment of cardiovascular risk factors. The reduction, particularly in hemorrhagic stroke, associated with increased exacerbations of COPD is hypothesis generating and also warrants further investigation into the care and treatment received by patients experiencing exacerbations in GP.

## Supplementary material

**Table S1:** Comparison of characteristics, between individuals with missing data and individuals without missing data

Characteristic	Number of individuals with missing smoking status (%), (n=41)	Number of individuals without missing smoking status (%), (n=25,723)	Number of individuals with missing GOLD stage (%), (n=3,492)	Number of individuals without missing GOLD stage (%), (n=22,272)	Number of individuals with missing BMI (%), (n=765)	Number of individuals without missing BMI (%), (n=24,999)
Stroke						
Yes	10 (24.4)	6,431 (25.0)	1,057 (30.3)	5,384 (24.2)	223 (29.2)	6,218 (24.9)
No	31 (75.6)	19,292 (75)	2,435 (69.7)	16,888 (75.8)	542 (75.0)	18,781 (75.1)
Exacerbations						
Infrequent	38 (92.7)	18,573 (72.2)	2,736 (78.4)	15,875 (71.3)	593 (77.5)	18,018 (72.1)
Frequent	3 (7.3)	7,150 (27.8)	756 (21.7)	6,397 (28.7)	172 (22.5)	6,981 (27.9)
Age (years)						
35–59	0 (0)	1,744 (6.8)	179 (5.1)	1,565 (7.0)	27 (3.5)	1,717 (6.9)
60–69	1 (2.4)	5,806 (22.6)	512 (14.7)	5,295 (23.8)	85 (11.1)	5,722 (22.9)
70–79	7 (17.1)	9,785 (38.0)	1,138 (32.6)	8,654 (38.9)	229 (29.9)	9,563 (38.3)
≥80	33 (80.5)	8,388 (32.6)	1,663 (47.6)	6,758 (30.3)	424 (55.4)	7,997 (40.0)
Sex						
Male	26 (63.4)	13,866 (53.9)	1,611 (46.1)	12,281 (55.1)	376 (49.2)	13,516 (54.1)
Female	15 (36.6)	11,857 (46.1)	1,881 (53.9)	9,991 (44.9)	389 (50.9)	11,483 (45.9)

**Abbreviations:** GOLD, Global Initiative on Obstructive Lung Disease; BMI, body mass index.

## Figures and Tables

**Table 1:** Baseline characteristics and univariable analysis of association with stroke

Characteristic	Cases (%), n=6,441	Controls (%), n=19,323	OR (95% CI)^a^
Male	3,473 (53.9)	10,419 (53.9)	
Age, mean years ± SD	74.9 (9.2)	74.8 (9.1)	
Frequent exacerbators	1,723 (26.8)	5,430 (28.1)	0.93 (0.87–0.99)
GOLD staging (n=22,272)			
1	880 (13.7)	2,639 (13.7)	1
2	2,777 (43.1)	8,351 (43.2)	0.98 (0.90–1.08)
3	1,407 (21.8)	4,754 (24.6)	0.87 (0.79–0.97)
4	320 (5.0)	1,144 (5.9)	0.82 (0.70–0.96)
Smoking status (n=25,723)			
Never smoked	627 (9.7)	2,062 (10.7)	1
Ex-smoker	2,776 (43.1)	8,806 (45.6)	1.04 (0.94–1.15)
Current smoker	3,028 (47)	8,424 (43.6)	1.20 (1.08–1.33)
Body mass index (n=24,999)			
<20	917 (14.8)	2,516 (13.4)	1
20–25	1,999 (31.2)	5,964 (31.8)	0.92 (0.84–1.01)
25–30	1,948 (31.3)	6,078 (31.5)	0.87 (0.80–0.96)
>30	1,354 (21.8)	4,223 (22.5)	0.87 (0.79–0.96)
Stroke risk factors			
Positive family history	1,615 (25.1)	4,688 (24.3)	1.05 (0.98–1.12)
Dyslipidemia	1,374 (21.3)	3,810 (19.7)	1.12 (1.04–1.20)
Hypertension	3,448 (53.5)	9,374 (48.5)	1.24 (1.17–1.31)
Diabetes	1,157 (18.0)	2,951 (15.27)	1.22 (1.13–1.32)
Cardiovascular disease			
PCI	202 (3.1)	514 (2.7)	1.19 (1.01–1.40)
PAD	724 (11.2)	1,503 (7.8)	1.51 (1.37–1.66)
Acute coronary syndrome	50 (0.8)	113 (0.6)	1.33 (0.95–1.86)
MI	746 (11.6)	1,970 (10.2)	1.16 (1.06–1.26)
Heart failure	895 (13.9)	2,317 (12.0)	1.19 (1.09–1.30)
CABG	239 (3.7)	589 (3.1)	1.24 (1.06–1.44)
Angina	1,142 (17.8)	3,163 (16.4)	1.10 (1.02–1.19)
Atrial fibrillation	1,064 (16.5)	2,146 (11.1)	1.61 (1.48–1.75)
Cardiac medications			
β-blocker	2,170 (33.7)	5,240 (27.1)	1.38 (1.30–1.47)
ACE inhibitor	3,017 (46.8)	8,051 (41.7)	1.24 (1.17–1.32)
Angiotensin II inhibitor	1,076 (16.7)	2,732 (14.1)	1.23 (1.14–1.33)
Nitrate	1,338 (20.7)	3,717 (19.2)	1.10 (1.03–1.18)
Calcium channel blocker	2,174 (33.8)	5,752 (29.8)	1.21 (1.14–1.29)
Lipid lowering medication	3,217 (50.0)	8,503 (44)	1.30 (1.22–1.37)
Antiplatelet	3,831 (59.5)	8,782 (45.5)	1.82 (1.71–1.93)
Anticoagulant	847 (13.2)	2,137 (11.1)	1.22 (1.12–1.32)

**Note:**

a Adjusted for age, sex, and GP.

**Abbreviations:** OR, odds ratio; CI, confidence interval; SD, standard deviation; GOLD, Global Initiative on Obstructive Lung Disease; PCI, percutaneous coronary intervention; PAD, peripheral arterial disease; MI, myocardial infarction; CABG, Coronary Artery Bypass Graft; ACE, angiotensin-converting enzyme; GP, general practice.

**Table 2:** Multivariable analysis of the odds of stroke and type of stroke, comparing COPD patients with frequent exacerbations to those with infrequent exacerbations

Stroke type	Exacerbation frequency	Cases (%)	Controls (%)	Crude OR^a^ (95% CI)	Adjusted OR^b^ (95% CI)
All stroke		**n**=**6,431**	**n**=**19,292**		
	Infrequent	4,709 (73.2)	13,864 (71.9)	1	1
	Frequent	1,722 (26.8)	5,428 (28.1)	0.94 (0.88–1.00)	0.95 (0.89–1.01)
Ischemic		**n**=**1,262**	**n**=**3,786**		
	Infrequent	944 (74.8)	2,769 (73.1)	1	1
	Frequent	318 (25.2)	1,017 (26.9)	0.93 (0.79–1.07)	0.93 (0.81–1.08)
Hemorrhagic		**n**=**420**	**n**=**1,260**		
	Infrequent	331 (78.8)	900 (71.4)	1	1
	Frequent	89 (21.2)	360 (28.6)	0.68 (0.52–0.88)	0.67 (0.51–0.88)
TIA		**n**=**2,457**	**n**=**7,371**		
	Infrequent	1,758 (71.6)	5,254 (71.3)	1	1
	Frequent	699 (28.5)	2,117 (28.7)	0.99 (0.89–1.09)	1.00 (0.90–1.11)

**Notes:**

a Adjusted for age, sex, GP, and smoking status,

b adjusted for age, sex, GP, smoking status, hypertension, diabetes, dyslipidemia, atrial fibrillation, heart failure, peripheral arterial disease, CABG, calcium channel blockers, β-blockers.

**Abbreviations:** COPD, chronic obstructive pulmonary disease; OR, odds ratio; CI, confidence interval; TIA, transient ischemic attack; GP, general practice; CABG, coronary artery bypass graft.
